# Hospital Expenditure.—The Commissariat

**Published:** 1895-11-16

**Authors:** 


### The Institutional Workshop.

### HOSPITAL EXPENDITURE.—THE COMMISSARIAT.

### VI.—THE RESIDENT MEDICAL OFFICERS.

The number of medical officers boarded in any hospital is insignificant compared with the number of patients or the size of the nursing staff. It does not for that reason follow that it forms an unimportant element of the total population from the housekeeper's point of view. What this element exactly amounts to as regards cost of maintenance it is not very easy in the case of most institutions to determine, since it is by no means the rule to check expenditure to the extent of ascertaining precisely the cost of one member of the household as distinguished from another. It is a common argument that when money is carefully spent, all waste avoided, and expenses cut down to a minimum, it is not the slightest advantage to know that more money is spent in one quarter than in another; but nothing can be more fallacious. In housekeeping of this complex description all knowledge is power, and no institution can be said to have a firm grasp of the financial reins unless the weekly cost of every class of inmates, within narrow limits of variation, has been accurately worked out, and is frequently tested.

In certain London hospitals the cost of provisions for the resident medical officers is separated from the sum total in the annual report, and it is possible to estimate the average weekly expenditure per head, and the percentage of this cost to the total spent on provisions. It is tempting to draw conclusions from those hospitals where the figures are given to those where no attempt has been ever made to estimate the cost, but nothing could be more dangerous than to attempt to draw inferences of this nature. Where facts are not forthcoming, guesses are worse than useless, and the following easily verified figures alone are given in the hope that they may stimulate inquiry into this subject among those who have the requisite data under their hands.

At St. Thomas's, in 1893, the board of ten medical officers figures in the report at £1,039, against a total of £9,935 for the whole establishment. This works out at an average of something under £2 a week per head, and the total is 10 per cent. per annum of the cost of food for the entire hospital, containing, on an average, 365 patients and close on 200 other inmates per day.

At Middlesex the board of nine medical officers, the secretary and chaplain, with several others lunching only, reaches a total of £885, at an average cost of from 25s. to 30s. a week pe head. This total is slightly under 12 per cent. of the cost of provisions, the hospital containing daily an average of 215 patients, plus 139 other inmates.

At Guy's the resident staff, consisting of four house-physicians, four house-surgeons, two obstetric residents, and two dressers for the week, are boarded by an arrangement with the committee of Guy's Hospital College. The total amount expended in 1893 on their provisions was £650, or a trifle over £1 a week per head, and this total amounted to no more than 6 per cent. of the cost of provisioning the whole establishment.

At Westminster five medical officers have full board, three house-surgeons partial board (everything except breakfast), and five other officials receive lunch and tea under a separate account, amounting in 1893 to £466. It is difficult to estimate at all accurately the weekly average per head, as partial board works out very differently under different circumstances. The cost of boarding the medical officers is 11 per cent. of the total cost of provisioning the hospital.

At University Hospital nine resident medical officers are boarded at the rate of £11 11s. a-week. The total expenditure on their provisions for the year is £600, and this reaches the extraordinary proportion of 17 per cent. of the cost of all the provisions consumed. It must be remembered, however, that no nurses are boarded in the hospital, and that the total sum expended on provisions is therefore relatively a good deal below the average.

At the Royal Free the cost of the medical staff is about a guinea a week, and the total on four receiving full board and one partial amounts to 8 per cent. of the total cost of provisions.

These examples are sufficient to prove that a wide difference in the cost of boarding this section of the hospital household is not incompatible with a very high general average. The number and cost of the doctors affect the average cost of the bed in a very striking manner. The average cost of the provisions per bed in Middlesex Hospital, for instance, would, if the board of doctors were reckoned in the total, rise from £30½ to £34½. If the doctors were not reckoned the average cost per bed would sink at Guy's Hospital from £24¾ to £23, and at University from £18½ to £15¼. It will be apparent, then, that a hospital like St. Bartholomew's, where the medical officers receive neither board nor allowance for board, should show a perceptible decrease in the average for provisions as compared with others.

The number of doctors boarded varies in London hospitals from fifteen at the London down to two or three at the smaller hospitals. The higher number generally works out at a lower percentage per bed. For instance, at the London the number of medical officers boarded is about in the proportion of two to every 100 beds, whereas this proportion is more than doubled in hospitals with two house surgeons and under fifty beds.

Outside London the highest number of doctors boarded is at Edinburgh Royal Infirmary with 14, Glasgow Royal Infirmary with 13, Glasgow West Infirmary with 11, Leeds General 11, and Birmingham General and Newcastle Royal Infirmary with 9. Two or three is the ordinary number for non-clinical hospitals in the country, and the arrangement for board in these is, as will be shown, rests on such an entirely different basis, that they do not bear any comparison with the system prevailing in London.

*** We are requested to point out that, owing to a double outbreak of diphtheria at the North-Western Hospital in 1893, necessitating twice the closing of the wards, the average number of occupied beds was less than usual, and the number of officials and rate of expenditure proportionately higher.

